# GNE-493 inhibits prostate cancer cell growth via Akt-mTOR-dependent and -independent mechanisms

**DOI:** 10.1038/s41420-022-00911-y

**Published:** 2022-03-16

**Authors:** Lu Jin, Wei Zhang, Ming-Yu Yao, Ye Tian, Bo-xin Xue, Wei Tao

**Affiliations:** 1 Department of Urology, the Second Affiliated Hospital of Soochow University, Suzhou, China; 2 Department of Radiology, the Second Affiliated Hospital of Soochow University Suzhou, Suzhou, China; 3 Department of Radiotherapy and Oncology, the Second Affiliated Hospital of Soochow University, Suzhou, China

**Keywords:** Targeted therapies, Prostate cancer

## Abstract

GNE-493 is a novel PI3K/mTOR dual inhibitor with improved metabolic stability, oral bioavailability, and excellent pharmacokinetic parameters. Here GNE-493 potently inhibited viability, proliferation, and migration in different primary and established (LNCaP and PC-3 lines) prostate cancer cells, and provoking apoptosis. GNE-493 blocked Akt-mTOR activation in primary human prostate cancer cells. A constitutively-active mutant Akt1 restored Akt-mTOR activation but only partially ameliorated GNE-493-induced prostate cancer cell death. Moreover, GNE-493 was still cytotoxic in Akt1/2-silenced primary prostate cancer cells. Significant oxidative stress and programmed necrosis cascade activation were detected in GNE-493-treated prostate cancer cells. Moreover, GNE-493 downregulated Sphingosine Kinase 1 (SphK1), causing ceramide accumulation in primary prostate cancer cells. Daily single dose GNE-493 oral administration robustly inhibited the growth of the prostate cancer xenograft in the nude mice. Akt-mTOR inactivation, SphK1 downregulation, ceramide level increase, and oxidative injury were detected in GNE-493-treated prostate cancer xenograft tissues. Together, GNE-493 inhibited prostate cancer cell growth possibly through the Akt-mTOR-dependent and -independent mechanisms.

## Introduction

Prostate cancer is the prostate epithelial malignancy affecting male populations [1, 2]. Even with the latest development of prostate-specific antigen (PSA) screening, it still accounts for a considerable proportion of global cancer-related mortalities [1, 2]. Radiotherapy and surgery remain to be the primary curative therapies in the management of localized prostate cancer [3–6].

PI3K-Akt-mTOR inhibitors have yielded promising anti-prostate cancer results [5, 7, 8]. Two distinct mTOR complexes have been identified: mTORC1 and mTORC2 [9, 10]. mTORC1 is responsible for phosphorylating S6K1 (p70S6 kinase), 4E-binding protein 1, and several others [10, 11]. mTORC2 functions as an upstream kinase for Akt (at the Ser-473 residue) and the other AGCs [10, 11].

Both mTORC1 and mTORC2 are required for the development and progression of prostate cancer [5, 7, 8]. Guertin et al. found that mTORC2 activation is important for PTEN loss-induced development of prostate cancer in mice [12]. Jiang et al. reported that a mTORC1/2 dual inhibitor INK-128 impeded prostate cancer cell growth [13]. Rapamycin (and its analogs) could partly suppress the activation of mTORC1, showing no direct effect on mTORC2 [14, 15]. Moreover, mTORC1 inhibition could induce feedback and sustained activation of Akt and Erk, both are pro-cancerous cascades [16, 17]. The ATP-competitive mTOR inhibitors were developed. These mTOR kinase inhibitors can block mTORC1 and mTORC2 simultaneously, often resulting better and more dramatic anti-cancer results [18].

GNE-493 is a novel PI3K-mTOR dual inhibitor [18]. GNE-493 showed improved metabolic stability, oral bioavailability, and good pharmacokinetic (PK) parameters [18]. The novel dual inhibitor could block the PI3K-Akt-mTOR cascade and inhibit breast xenograft growth in mice [18]. GNE-493’s potential effect on prostate cancer cell growth was tested here.

## Results

### GNE-493 exerts tumor-suppressive activity in cultured prostate cancer cells

**Fig. 1:**
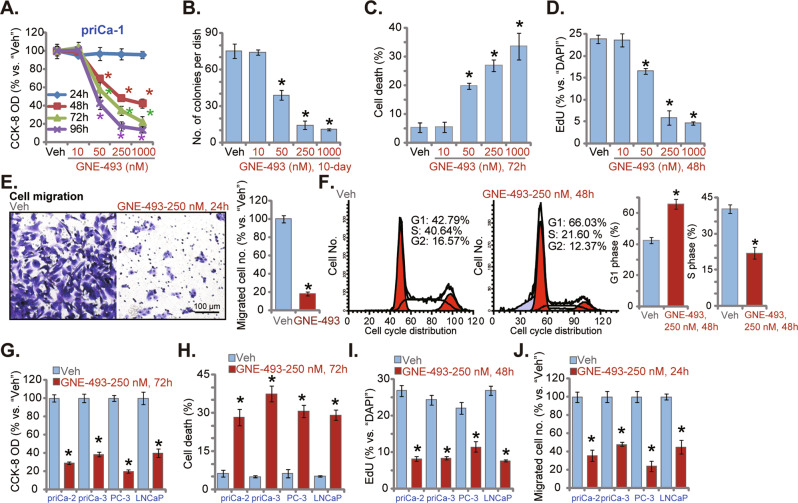
GNE-493 exerts tumor-suppressive activity in cultured prostate cancer cells. The patient-derived primary prostate cancer cells (“priCa-1/-2/-3”, that were derived from three patients) or established cell lines (PC-3 or LNCaP) were cultivated in complete medium and stimulated with the applied concentrations of GNE-493 (10–1000 nM) or the vehicle control (0.25% DMSO, “Veh”); cells were further cultivated for a designated time, cell viability (by measuring CCK-8 OD, **A** and **G**), the number of cell colonies (**B**), cell death (by recording the Trypan blue percentage, **C** and **H**), and cell proliferation (by recording the EdU-positively stained nuclei percentage, **D** and **I**) as well as the in vitro cell migration (“Transwell” assays, **E** and **J**) and distribution of cell cycles (PI-FACS assays, **F**) were tested. *P < 0.05 versus “Veh” group. Scale bar = 100 μm (**E**).

The primary human prostate cancer priCa-1cells were cultivated in complete medium and treated with GNE-493 from 10 to 1000 nM. GNE-493 dose-dependently decreased priCa-1 cell viability (CCK-8 OD) (Fig. 1A). Viability reduction was significant after 50–1000 nM of GNE-493 treatment (Fig. 1A), but it was insignificant at 10 nM (Fig. 1A). GNE-493-induced viability reduction in priCa-1 cells was time-dependent (Fig. 1A). Figure 1B showed that the viable priCa-1 cell colonies were significantly reduced after GNE-493 (50–1000 nM) treatment. Moreover, GNE-493 dose-dependently increased the ratio of Trypan blue-positive (“dead”) priCa-1 cells (Fig. 1C). Furthermore, GNE-493 (50–1000 nM) significantly decreased the EdU positively incorporated nuclei ratio in priCa-1 cells (Fig. 1D), supporting its anti-proliferative activity. As shown, 10 nM of GNE-493 failed to significantly affect colony number (Fig. 1B), cell death (Fig. 1C), and nuclear EdU incorporation (Fig. 1D) in priCa-1 primary cells. These titration experiment results (Fig. 1A–D) showed that 250 nM of GNE-493 exerted significant tumor-suppressive activity. We selected this concentration for the following experiments.

“Transwell” assay results showed that GNE-493 (250 nM for 24 h) robustly decreased the number of migration (Fig. 1E) priCa-1 cells. The PI-FACS assay results showed that GNE-493 (250 nM, 48 h) disrupted cell cycle progression in priCa-1 cells, causing increased G1-phase cell percentage but decreased S-phase cell percentage (Fig. 1F).

Other prostate cancer cells, including the primary human prostate cancer cells of two other patients, priCa-2 and priCa-3, as well as the immortalized prostate cancer cell lines, LNCaP and PC-3, were also treated with GNE-493 (250 nM). The PI3K-mTOR dual inhibitor robustly inhibited CCK-8 cell viability (Fig. 1G) and induced significant cell death (Fig. 1H) in the prostate cancer cells. In addition, GNE-493 (250 nM) robustly inhibited proliferation (tested by the decreased ratio of EdU-incorporated nuclei, Fig. 1I) and migration (Fig. 1J) of the primary and immortalized prostate cancer cells.

### GNE-493 provokes apoptosis in prostate cancer cells

**Fig. 2:**
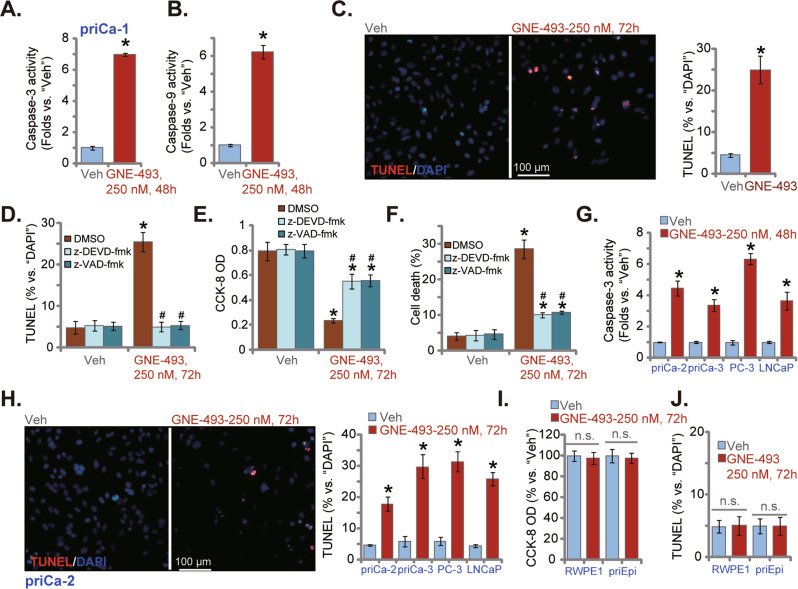
GNE-493 provokes apoptosis in prostate cancer cells. The patient-derived primary prostate cancer cells (priCa-1/-2/-3) or the established cell lines (PC-3 cells or LNCaP) were cultivated in complete medium and stimulated with GNE-493 (250 nM) or the vehicle control (0.25% DMSO, “Veh”); cells were further cultivated for designated time, the caspase-3/caspase-9 activities were tested (**A**, **B** and **G**). Cell apoptosis was examined by the nuclear TUNEL staining assay (**C** and **H**). The priCa-1 primary prostate cancer cells were pretreated with z-DEVD-fmk, z-VAD-fmk (for 30 min, each at 50 μM) or the vehicle control (0.25% DMSO), cells were further treated with GNE-493 (250 nM) and cultivated for designated time, cell apoptosis, cell viability, and death were tested by TUNEL staining (**D**), CCK-8 (**E**) and Trypan blue staining (**F**) assays, respectively. The established Prostate epithelial RWPE1 cells or primary prostate epithelial cells (“priEpi”) were treated with GNE-493 (250 nM) or the vehicle control (0.25% DMSO, “Veh”), and were cultivated for 72 h; testing cell viability and apoptosis were through CCK-8 (**I**) and nuclear TUNEL staining (**J**) assays, respectively. *P < 0.05 versus “Veh” group. ^#^*P* < 0.05 versus GNE-493 treatment (**D**–**F**). “n.s.” stands for non-statistical difference (**I** and **J**). Scale bar = 100 μm (**C** and **H**).

We next tested whether GNE-493 could induce apoptosis in human prostate cancer cells. The relative caspase-3 and caspase-9 activities (Fig. 2A, B) were significantly increased in GNE-493 (250 nM, 48 h)-treated priCa-1 cells. Following GNE-493 treatment, the TUNEL positively stained nuclei ratio was increased significantly in priCa-1 cells, indicating apoptosis activation (Fig. 2C). Significantly z-DEVD-fmk (an established caspase-3 specific inhibitor) and z-VAD-fmk (a pan caspase inhibitor) blocked GNE-493-induced apoptosis (TUNEL staining assays) in priCa-1 primary cancer cells (Fig. 2D). The two caspase inhibitors largely attenuated GNE-493-caused viability decrease (Fig. 2E) and priCa-1 cell death (Fig. 2F).

In other primary prostate cancer cells (priCa-2 and priCa-3) and immortalized cell lines (LNCaP and PC-3), treatment with GNE-493 (250 nM) significantly increased the relative caspase-3 activity (Fig. 2G). In addition, the TUNEL positively stained nuclei ratio (Fig. 2H) was significantly increased, confirming apoptosis activation. Contrarily, in the immortalized prostate epithelial RWPE1 cells and the primary human prostate epithelial cells (“priEpi”), GNE-493 (250 nM) treatment did not induce viability decrease (Fig. 2I) and apoptosis (TUNEL staining assays, Fig. 2J).

### GNE-493 blocks Akt-mTOR activation in prostate cancer cells

**Fig. 3:**
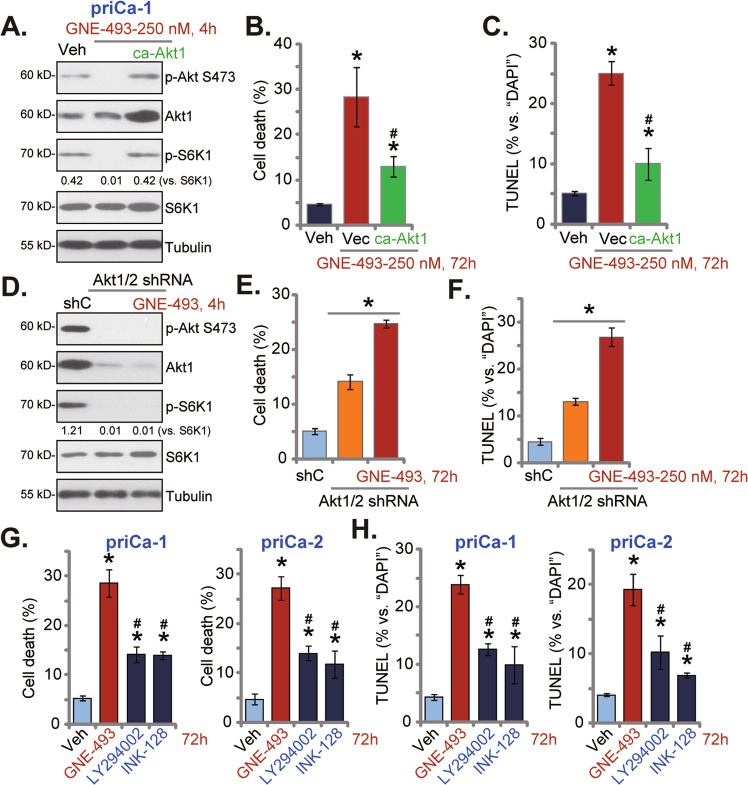
GNE-493 blocks Akt-mTOR activation in prostate cancer cells. The priCa-1 cells were infected the recombinant adenovirus encoding the constitutively-active mutant Akt1 (caAkt1, S473D, for 72 h), followed by GNE-493 (250 nM) stimulation for designated time, control cells were treated with vehicle control; western blotting assays showed expression of listed proteins n (**A**); cell death (by measuring Trypan blue-positive cell percentage, **B**) and cell apoptosis (by measuring the TUNEL-positive nuclei percentage, **C**) were tested. priCa-1 cells stably expressing the lentiviral Akt1/2 shRNA (Akt1/2 shRNA, for 72 h) were stimulated with or without GNE-493 (250 nM); control shRNA lentiviral particles (“shC”) were added to the control cells. Cells were further cultivated for designated time, listed proteins were shown (**D**); cell death (by measuring Trypan blue-positive cell percentage, **E**) and apoptosis (by measuring TUNEL-positive nuclei percentage, **F**) were tested. The primary human prostate cancer cells, priCa-1 or priCa-2, were treated with 250 nM of GNE-493, LY294002, INK-128, or the vehicle control (0.25% DMSO, “Veh”) and cells were cultivated for 72 h, cell death (**G**) and cell apoptosis (**H**) were tested similarly. *P < 0.05 versus “Veh” group (**B**, **C**, **G**, and **H**). ^#^*P* < 0.05 versus “Vec” cells (**B** and **C**). *P < 0.05 (**E** and **F**). ^#^*P* < 0.05 versus GNE-493 treatment (**G** and **H**).

In priCa-1 primary cancer cells, GNE-493 treatment (250 nM, 4 h) blocked phosphorylations of S6K1 (at the Thr-389 residue) and Akt (at the Ser-473 residue), confirming Akt-mTOR cascade blockage (Fig. 3A). The constitutively-active mutant Akt1 (ca-Akt1, S473D) viral construct was stably transduced to priCa-1 cells, and it completely recovered Akt-S6K1 phosphorylations in GNE-493-treated priCa-1 cells (Fig. 3A). The ca-Akt1 yet partly ameliorated GNE-493-induced cell death (Fig. 3B) and induction of apoptosis (by measuring TUNEL ratio, Fig. 3C) in priCa-1 cells.

The lentiviral particles encoding the Akt1/2 shRNA sequence were transfected to priCa-1 cells. The depleted Akt1/2 expression was detected in stable cells (Fig. 3D). S6K1 and Akt phosphorylations were blocked in Akt1/2-depleted priCa-1 cells (Fig. 3D). Although Akt1/2 silencing induced moderate cell death (Fig. 3E) and apoptosis (Fig. 3F) in priCa-1 cells, GNE-493 was capable of inducing further cytotoxicity (Fig. 3E, F). Therefore, the Akt-mTOR-independent mechanisms could possibly participate in GNE-493-induced cytotoxicity in prostate cancer cells as well. Moreover, GNE-493 (250 nM)-induced death (Fig. 3G) and apoptosis (Fig. 3H) in primary prostate cancer cells were more potent than the same concentration of LY294002 (the pan PI3K-Akt-mTOR inhibitor [19]) or INK-128 (the mTOR kinase inhibitor [13]) in priCa-1 or priCa-2 primary cancer cells.

### GNE-493 induces oxidative stress and programmed necrosis in prostate cancer cells

**Fig. 4:**
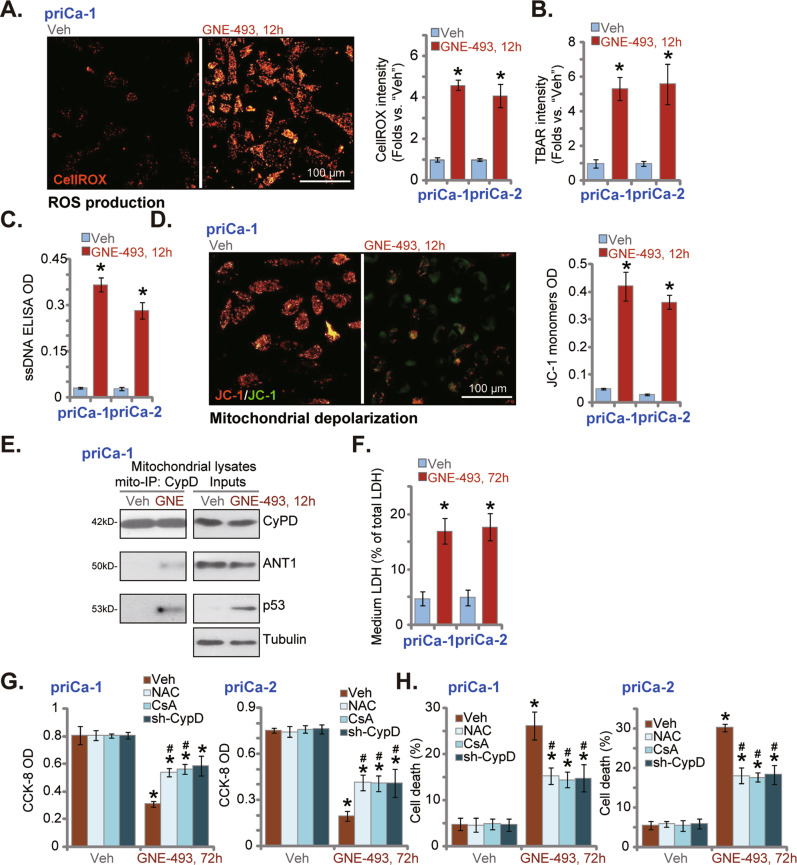
GNE-493 induces oxidative stress and programmed necrosis in prostate cancer cells. The primary prostate cancer cells (priCa-1 or priCa-2) were stimulated with GNE-493 (250 nM) or the vehicle control (0.25% DMSO, “Veh”); cells were further cultivated for designated time, ROS contents (by examining CellROX intensity, **A**), lipid peroxidation (by examining TBAR intensity, **B**), DNA break (by examining ssDNA contents, **C**) and mitochondrial depolarization (tested by green JC-1 monomers accumulation, **D**) were tested. Mitochondrial immunoprecipitation (“Mito-IP”) assays were employed to examine the association of p53-CyPD-ANT1 (in priCa-1 cells, **E**); expression of p53-CyPD-ANT in mitochondrial lysates was tested by Western blotting assays (**E**, “Inputs”). Cell necrosis was tested by measuring medium LDH release (**F**). The primary human prostate cancer cells, priCa-1 or priCa-2, were pretreated for 30 min with N-acetylcysteine (NAC, 400 μM) or the CyPD inhibitor cyclosporin A (CsA, 10 μM), or stably infected with CyPD shRNA lentivirus (sh-CyPD), followed by GNE-493 (250 nM) treatment and cultivated for 72 h, cell viability and cell death were then separately examined by CCK-8 (**G**) and the Trypan blue staining (**H**) assays. *P < 0.05 versus “Veh” group. ^#^*P* < 0.05 versus “GNE-493” only treatment. Scale bar = 100 μm (**A** and **D**).

Although GNE-493 provoked apoptosis activation in prostate cancer cells, the caspase inhibitors failed to completely reverse GNE-493-induced cell death (see Fig. 2). Recent studies have proposed an active and “programmed” mitochondrial necrosis cascade, or programmed necrosis. It is essential in mediating cancer cell death by a number of anticancer agents and various other stimuli [20–29]. Different cancer-killing agents could induce robust ROS production and significant oxidative injury, leading to p53 protein translocation to mitochondrion and association with CyPD and ANT1. The formation of this mitochondrial p53-CyPD-ANT1 complex will then induce the opening of mPTP channel (mitochondrial permeability transition pore) and depolarization of mitochondria, and eventually causing cell necrosis [22, 24, 26, 30, 31]. By employing a CellROX staining assay, we found that levels of ROS (by measuring the CellROX intensity) were significantly increased in GNE-493-treated priCa-1 and priCa-2 primary cancer cells (Fig. 4A). Significant lipid peroxidation, reflected by the increase of the TBAR activity, was observed as well (Fig. 4B). Moreover, ssDNA accumulation was detected after GNE-493 treatment, reflecting increased DNA damage in priCa-1 and priCa-2 primary cancer cells (Fig. 4C).

Significant mitochondrial depolarization was detected in GNE-493-treated primary prostate cancer cells as well, as it induced the mitochondrial accumulation of JC-1 monomers (green fluorescence, Fig. 4D). The mitochondrial immunoprecipitation experimental results showed that the CyPD protein immunoprecipitated with p53 and ANT1 in response to GNE-493 treatment in priCa-1 cells (Fig. 4E). “Inputs” assays confirmed p53 translocation to mitochondria after GNE-493 treatment in cancer cells (Fig. 4E, “Inputs”). CyPD and ANT1 expression was unchanged (Fig. 4E, “Inputs”). Significantly, the medium LDH contents were increased in GNE-493-treated primary cancer cells (Fig. 4F), supporting cell necrosis induction. Therefore, GNE-493 induced significant oxidative injury and programmed necrosis cascade in prostate cancer cells.

To block oxidative injury the ROS scavenger N-acetyl-L-cysteine (NAC), the CyPD inhibitor cyclosporin A (CsA) [21, 23, 24, 32], and CyPD shRNA lentivirus were employed. In both priCa-1 and priCa-2 primary cancer cells, GNE-493-induced viability reduction (CCK-8 assays, Fig. 4G) and cell death (Fig. 4H) were partially attenuated by NAC, CsA, and CyPD shRNA. Therefore, oxidative stress and programmed necrosis cascade also participated in GNE-493-induced prostate cancer cell death.

### GNE-493 downregulates SphK1 protein and induces ceramide accumulation in prostate cancer cells

**Fig. 5:**
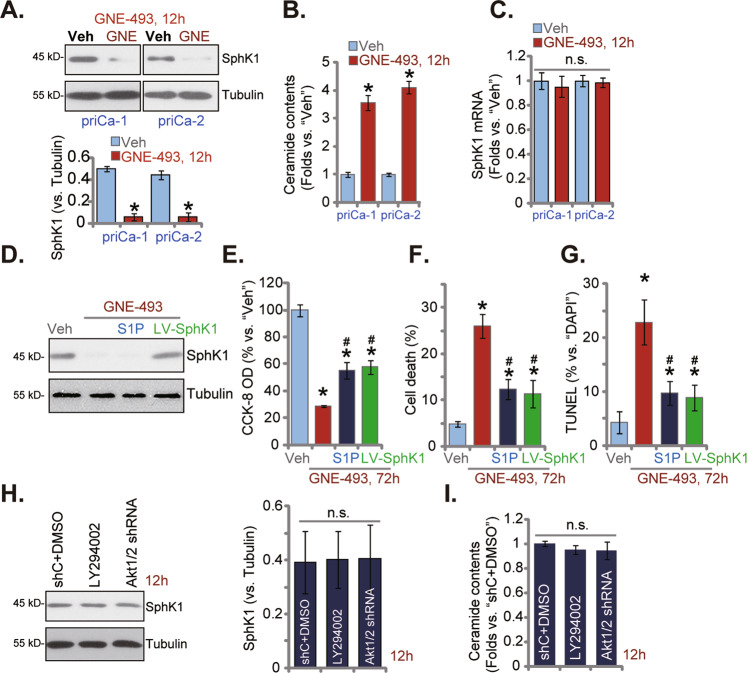
GNE-493 downregulates SphK1 protein and induces ceramide accumulation in prostate cancer cells. The primary prostate cancer cells, priCa-1 or priCa-2, were stimulated with GNE-493 (250 nM) or the vehicle control (0.25% DMSO, “Veh”); cells were further cultivated for an additional 12 h, the SphK1 protein expression (**A**), the ceramide contents (**B**) and the *SphK1* mRNA expression (**C**) were tested. priCa-1 cells were infected with SphK1-expressing lentiviral particles (LV-SphK1, for 72 h) or pretreated with sphingosine 1-phosphate (S1P, 10 μM, 2 h), followed by GNE-493 (250 nM) treatment, control cells were treated with vehicle control. Cells were further cultivated for 72 h, cell viability (by testing the CCK-8 OD, **E**), cell death (by measuring the Trypan blue-positive cell percentage, **F**) and apoptosis (by examining the TUNEL-positive nuclei percentage, **G**) were tested. priCa-1 cells were infected with Akt1/2 shRNA lentiviral particles (Akt1/2 shRNA, for 72 h) or treated with LY294002 (1 μM), the control cells were treated with the control shRNA lentiviral particles (“shC”) plus DMSO (0.1%); Cells were cultivated for additional 12 h, levels of SphK1 protein (**H**) and ceramide contents (**I**) were tested. *P < 0.05 versus “Veh” group. ^#^*P* < 0.05 versus “GNE-493” only treatment. “n.s.” stands for non-statistical difference.

SphK1 is a key player in carcinogenesis and the development of prostate cancer. In priCa-1 and priCa-2 primary cancer cells, SphK1 protein levels were dramatically decreased after GNE-493 treatment (250 nM, 12 h) (Fig. 5A). Ceramide levels were significantly increased (Fig. 5B). *SphK1* mRNA expression was unchanged following GNE-493 treatment (Fig. 5C). Next, a lentiviral SphK1-expressing construct (“LV-SphK1”) was transduced to priCa-1 cells, which restored SphK1 expression in GNE-493-treated priCa-1 cells (Fig. 5D). Alternatively, the anti-ceramide lipid sphingosine 1-phosphate (S1P) was utilized. As shown GNE-493-induced CCK-8 viability decrease (Fig. 5E), cell death (Fig. 5F), and cell apoptosis (Fig. 5G) were partly attenuated by LV-SphK1 and S1P. These results implied that SphK1 downregulation participated in GNE-493-induced cytotoxicity in prostate cancer cells. Notably, in priCa-1 cells, Akt1/2 shRNA (see Fig. 4) or LY294002 (the pan PI3K-Akt-mTOR blocker) did not affect SphK1 expression (Fig. 5H). Nor did it altered ceramide contents (Fig. 5I). These results implied that SphK1 downregulation and ceramide accumulation were unlikely the consequence of Akt-mTOR blockage in GNE-493-treated prostate cancer cells.

### GNE-493 oral administration inhibits prostate cancer xenograft growth in nude mice

**Fig. 6:**
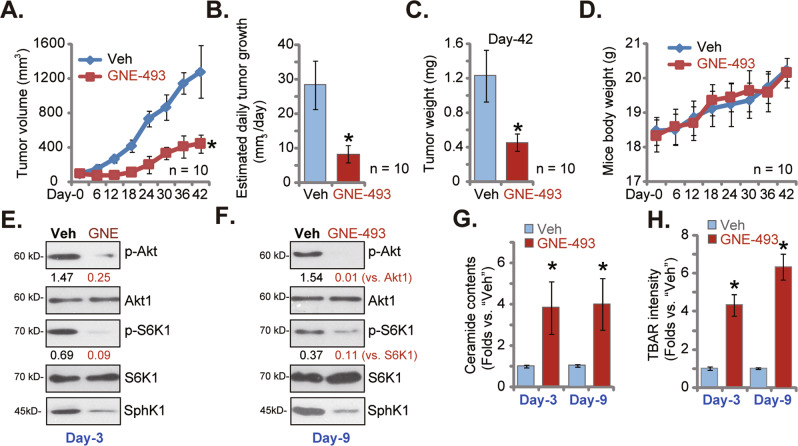
GNE-493 oral administration inhibits prostate cancer xenograft growth in nude mice. The priCa-1 xenograft tumor-bearing nude mice were subject to daily oral administration of a single dose of GNE-493 (20 mg/kg body weights, 12 days) or the vehicle control (“Veh”); volumes of xenograft tumors (**A**) and the animal body weights (**D**) were recorded; the estimated daily tumor growth was calculated as well (**B**); the xenografts were all carefully isolated and weighted at Day-42 (**C**). At experimental Day-3 and experimental Day-9, one tumor from each group was isolated 6 h after initial GNE-493 or vehicle administration. In the tumor tissues listed proteins were examined (**E** and **F**). Total ceramide contents (**G**) and the relative TBAR activity (**H**) were examined as well. *P < 0.05 versus “Veh” group.

As described the nude mice bearing priCa-1 xenografts, ten mice per group, were subject to oral administration of GNE-493 (at 20 mg/kg body weights), daily for 12 days or the vehicle control (“Veh”). Figure 6A showed that in nude mice GNE-493 administration potently inhibited growth of the priCa-1 xenograft tumors. The described formula [33] was utilized to calculate the estimated daily tumor growth and results found that priCa-1 xenograft tumor growth was robustly inhibited after GNE-493 (Fig. 6B). At Day-42, all tumors were carefully isolated and tumor weights were recorded. As shown in GNE-493 treatment group the xenograft tumors were lighter than those-treated with vehicle control (Fig. 6C). No significant difference in animal body weights was detected among the treatment and vehicle control groups (Fig. 6D). Thus, a daily single dose of GNE-493 administration largely inhibited priCa-1 xenograft growth in nude mice.

At Day-3 and Day-9, 6 h after initial GNE-493 or vehicle administration one tumor from each group was separated carefully. As shown, S6K1 and Akt phosphorylations were largely inhibited in GNE-493-treated priCa-1 xenograft tissues (Fig. 6E, F). SphK1 protein levels were decreased (Fig. 6E, F), while the total ceramide levels (Fig. 6G) were increased in GNE-493-treated tumor tissues. In addition, TBAR activity was significantly enhanced in GNE-493-treated tumor tissues (Fig. 6H), supporting oxidative injury response. Therefore GNE-493 oral administration blocked Akt-mTOR signaling, downregulated SphK1, induced ceramide accumulation, and oxidative injury in priCa-1 xenograft tissues.

## Discussion

Due to PTEN depletion, PIK3CA amplification, and other genetic changes, the PI3K-Akt-mTOR pathway is most frequently overactivated in prostate cancer [5, 7, 8], which is associated with cancer progression, cancer metastasis, as well as development of drug resistance [5, 7, 8]. Preclinical studies examining specific PI3K-Akt-mTOR inhibitors have yielded promising results, yet the results of the clinical trials are less convincing [5, 7, 8]. Emerging studies have suggested that dual PI3K-mTOR inhibitors, which can block the entire PI3K-Akt-mTOR cascade, could achieve better and more robust anti-cancer efficiency [5, 7, 8].

Here we showed that GNE-493 efficiently inhibited prostate cancer cell growth. In multiple primary prostate cancer cells and established cell lines (LNCaP and PC-3), GNE-493 robustly inhibited cell viability, proliferation, and in vitro migration, while inducing cell cycle arrest and provoking apoptosis. GNE-493 blocked Akt-mTOR activation in prostate cancer cells. In vivo studies showed that a daily single dose of GNE-493 administration significantly inhibited prostate cancer xenograft growth in nude mice. In GNE-493-treated prostate cancer xenografts, Akt-mTOR inactivation was also detected.

Akt-mTOR blockage however is not the sole mechanism responsible for prostate cancer cell death by GNE-493. caAkt1 reserved Akt-S6K1 phosphorylations yet only partially attenuated GNE-493-induced prostate cancer cell death. GNE-493 was still cytotoxic and induced apoptosis in Akt1/2-silenced cancer cells. Indeed we discovered that Akt-mTOR-independent mechanisms by GNE-493, including ROS production and oxidative injury, programmed necrosis, SphK1 downregulation, and ceramide accumulation, were also important in mediating prostate cancer cell death.

A number of anti-cancer agents could provoke programmed necrosis cascade. Different stimuli will induce p53 mitochondrial translocation and association with CyPD and ANT-1 to open mPTP. This will lead to MMP reduction and finally cause cell necrosis [22, 24, 26, 30, 31]. PF-543, the known SphK1 inhibitor, activated programmed necrosis pathway in human colorectal cancer (CRC) cells [21]. AICAR activated AMPK-independent programmed necrosis cascade to cause death of prostate cancer cells [22]. Qin et al. found that glioma cell death by salinomycin was due to programmed necrosis, which can be inhibited by CyPD silencing or inhibition [23].

In addition, a number of oxidative stimuli could also provoke the programmed necrosis cascade [27, 34–36]. Here we discovered that GNE-493 provoked oxidative injury and programmed necrosis cascade in prostate cancer cells. Significant ROS production, increased lipid peroxidation, and accumulated DNA damage were detected in GNE-493-treated prostate cancer cells. Moreover, GNE-493 induced p53 translocation to mitochondria and p53-CyPD-ANT1 complex formation as well as mitochondrial depolarization and necrosis. Importantly, the antioxidant NAC, the CyPD inhibitor CsA as well as CyPD shRNA ameliorated GNE-493-induced cytotoxicity in prostate cancer cells. Therefore, besides apoptosis, oxidative stress and programmed necrosis are contributors of GNE-493-induced prostate cancer cell death.

SphK1 is overexpressed and/or overactivated in prostate cancer, serving as an important diagnosis marker and therapeutic target [37, 38]. It has been shown that circulating S1P contents and erythrocyte SphK1 activity could be novel and efficient biomarkers for the early detection of prostate cancer [39]. Sauer et al. reported that pharmacological inhibition or siRNA-mediated silencing of SphK1 sensitized hormone-resistant prostate cancer cells to docetaxel-induced apoptosis [40]. Pchejetski et al. reported that FTY720, a SphK1 inhibitor, induced prostate cancer cell apoptosis [41]. Dayon et al. showed that SphK1 activation upon chronic androgen deprivation was essential for prostate cancer cell growth and survival [42]. We here discovered that SphK1 protein downregulation and ceramide accumulation, independent of Akt-mTOR inactivation, were also important for prostate cancer cell death by GNE-493.

## Conclusion

GNE-493 inhibited prostate cancer cell growth possibly through the Akt-mTOR-dependent and -independent mechanisms

## Material and methods

### Chemicals and reagents

Puromycin, polybrene, antibiotics, medium, serum, JC-1, Cell Counting Kit-8 (CCK-8), INK-128, LY294002, TUNEL (Terminal deoxynucleotidyl transferase dUTP nick end labeling) dye, and CellROX dye were from Sigma-Aldrich (St. Louis, Mo). GNE-493 was obtained from Selleck (Beijing, China). Z-VAD-FMK and z-DVED-FMK were purchased from Merck Millipore (Shanghai, China). The ANT1 (adenine nucleotide translocase 1) antibody (catalog number ab102032) was provided by the Abcam Co.. Antibodies for Cyclophilin-D (CyPD, catalog number sc-137136) and p53 (catalog number sc-126) were from Santa Cruz Biotech. Other antibodies were described previously [20]. EdU, and DAPI were purchased from Invitrogen (Thermo-Fisher, Shanghai, China).

### Cell lines

The immortalized prostate cancer cell lines, LNCaP and PC-3, as well as RWPE1 immortalized prostate epithelial cells, were from Dr. Wang [13]. Cells were cultivated using the described protocols [13].

### Primary culture of patient-derived primary human prostate cancer cells

Three primary prostate cancer patients undergoing prostate resection, with the written-informed consent, were enrolled in this study. The prostate cancer tissues and adjacent surrounding normal prostate tissues were carefully separated. Tissues were minced and digested as described [13]. Primary prostate cancer cells or prostate epithelial cells were cultivated in medium descried [13]. Primary prostate cancer cells were derived from three patients, “priCa-1”, “priCa-2”, and “priCa-3”. The primary prostate epithelial cells were from one patient and were named as “priEpi” cells. This study and the protocols were approved by the Ethics Committee of The Second Affiliated Hospital of Soochow University and were in accordance with Declaration of Helsinki principles.

### Colony formation assay

The prostate cancer cells were treated as descried and re-suspended in complete medium with 0.5% agar (Sigma) and plated on the top of a 10-cm diameter culture dish. Every two days GNE-493-containing medium was renewed. Afterwards, the number of viable prostate cancer cell colonies were manually counted.

### FACS

Briefly, cells were fixed and stained with propidium iodide (PI, at 10 μg/mL) for 30 min. The FACS assays were carried out to examine cell cycle distribution.

### Other assays

Cells were seeded at optimal seeding conditions. The detailed protocols of Western blotting, quantitative real time-PCR (qRT-PCR), single strand DNA (ssDNA) detection, CCK-8 viability, Trypan blue cell death assay, caspase-3/-9 activity assays, the nuclear TUNEL staining, the JC-1 mitochondrial depolarization assay, nuclear EdU staining and “Transwell” assays were described previously [20]. mRNA primers for *Sphingosine Kinase 1* (*SphK1*) and GAPDH were described previously [43–45]. The uncropped blotting images were presented in Figure S1.

### Constitutively-active mutant Akt1

Prostate cancer cells were cultivated in the polybrene-containing complete medium (with FBS) and treated with the recombinant adenoviral constitutively-active mutant S473D Akt1 (caAkt1, from Dr. Li [46, 47]). Expression of caAkt1 in the infected cells was verified by Western blotting.

### Akt1/2 shRNA

Prostate cancer cells were cultivated in polybrene-containing medium (with FBS) and infected with the Akt1/2 shRNA lentiviral particles (catalog number sc-37030-V, Santa Cruz Biotech) for a total of 24 h. Afterwards, puromycin (3.5 μg/mL) was included the medium and stable cells were established.

Lipid peroxidation assay. Prostate cancer cells were initially seeded into the six-well plates at 8 × 10^4^ cells per well and subjected to the applied GNE-493 treatment. The lipid peroxidation levels in total cellular lysates and tissue lysates were measured and quantified using thiobarbituric acid reactive substances (TBAR) method according to the detailed protocols described [48, 49].

### Reactive oxygen species (ROS) detection

Prostate cancer cells were initially seeded onto the six-well plates at 8 × 10 ^4^ cells per well and subjected to applied GNE-493 treatment. Afterwards, cells were washed with cold PBS and stained with CellROX (3.5 μg/mL). CellROX fluorescence intensity, reflecting ROS contents, was detected by the fluorescence spectrofluorometer at 625 nm.

### Ceramide assay

Prostate cancer cells were subjected to applied GNE-493 treatment. Ceramide contents in total cellular lysates and tissue lysates were tested via the protocol as described previously [50].

### Mitochondrial immunoprecipitation (Mito-IP)

Mito-IP experiments were performed under the previously-described protocols [51, 52]. Briefly, the pre-cleared mitochondrial lysates were incubated with an anti-CyPD antibody (purchased from Santa Cruz Biotech). CyPD-immunoprecipitated proteins, ANT1 and p53, were captured and examined. Expression of ANT1, p53, and CyPD in mitochondrial fraction lysates was tested as “Inputs”.

### CyPD shRNA

In brief, the primary human prostate cancer cells were treated with CyPD shRNA lentiviral particles (Santa Cruz Biotech) for 48 h. Afterwards, to select stable cells puromycin (2.5 μg/mL) was added for another 4-5 days. CyPD silencing, with over 90–95% knockdown efficiency, was tested by Western blotting assays.

### SphK1 expression

A SphK1 over-expressing lentiviral construct was from Dr. Yao [53]. The SphK1-expressing construct, together with the lentivirus Helper plasmids, were co-transfected to HEK-293T cells. The SphK1 over-expressing lentivirus, LV-SphK1, was thereafter added to primary prostate cancer cells. SphK1 overexpression was always confirmed by Western blotting assays.

### Xenograft assay

The male nude mice, at 6–8 weeks old, 18.5–19.0 g, were purchased from the animal facility of The Second Affiliated Hospital of Soochow University, and maintained under the Institutional Animal Care Use Committee guidelines. priCa1 primary prostate cancer cells were mixed at 1:1 ratio with Matrigel. To the right flanks of the mice, six millions cells (in 100 μL suspension) were subcutaneously (*s.c*.) injected to each nude mice. Treatment began four weeks post pri-Ca1 cells implantation, and the volume of each tumor reached around 100 mm^3^. The xenograft bearing nude mice were orally administrated with GNE-493 (20 mg/kg) or the vehicle control daily for 12 consecutive days [54]. Measuring the tumor volumes, the mice body weights, and the tumor weights were determined as described [55]. To test signaling changes, tumor tissues were minced, homogenized, and dissolved in tissue lysis buffer. Protocols of handling the nude mice were reviewed and approved by the Institute Animal Ethics Review Board of The Second Affiliated Hospital of Soochow University.

### Statistical analyses

Data were mean ± standard deviation (S.D.). Statistical analyses were performed as described [20, 44].

## Supplementary information

Figure S1

## Data Availability

All data are available upon request.
