# Supplementary figures and images for: GNE-493 inhibits prostate cancer cell growth via Akt-mTOR-dependent and -independent mechanisms
Source: Cell Death Discov. 2022 Mar 16;8:120. doi: 10.1038/s41420-022-00911-y (PMC8927604; doi:10.1038/s41420-022-00911-y)

Figure S1: The uncropped blotting images of the study

Figure 3

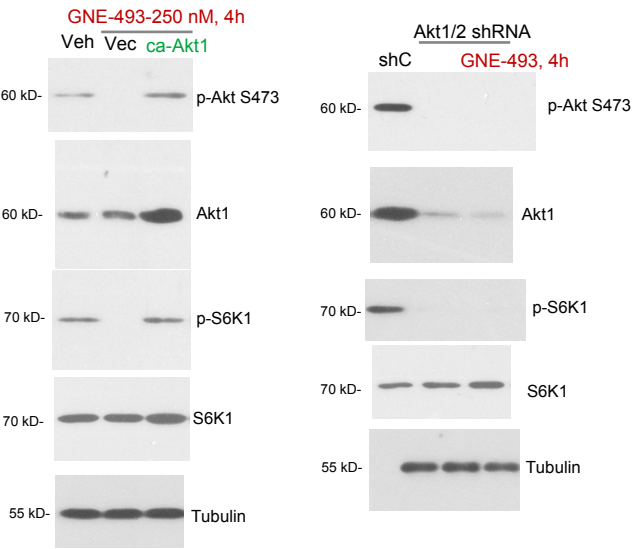

Figure 4

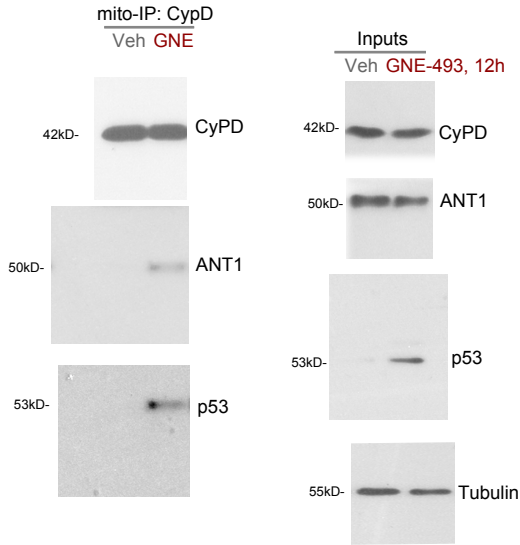

Figure 5.

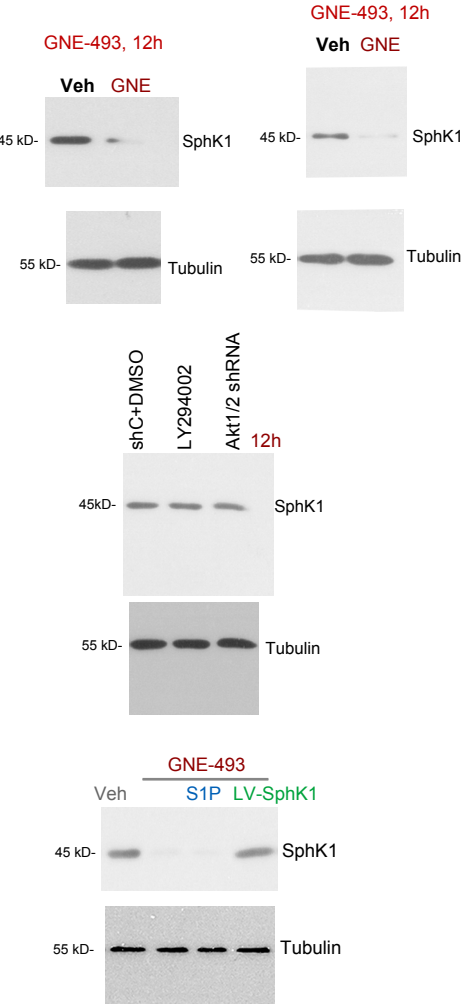

Figure 6.

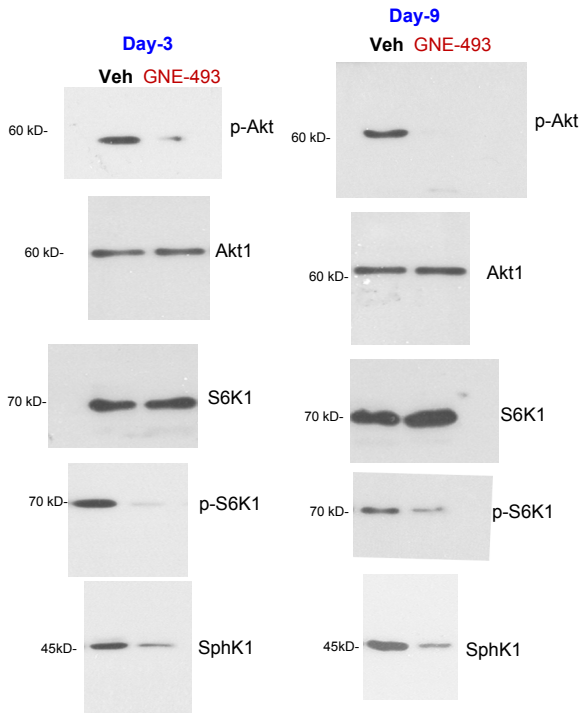

Supplement: Supplementary file 1 — Figure S1 [file 41420_2022_911_MOESM1_ESM.pdf]
